# Efficient optimization techniques for resource allocation in UAVs mission framework

**DOI:** 10.1371/journal.pone.0283923

**Published:** 2023-04-06

**Authors:** Sohail Razzaq, Costas Xydeas, Anzar Mahmood, Saeed Ahmed, Naeem Iqbal Ratyal, Jamshed Iqbal

**Affiliations:** 1 Faculty of Information Technology, Majan University College, Muscat, Sultanate of Oman; 2 Department of Electrical and Computer Engineering, COMSATS University Islamabad, Abbottabad Campus, Abbottabad, Pakistan; 3 School of Computing and Communications, Lancaster University, Lancaster, United Kingdom; 4 Department of Electrical Engineering, Mirpur University of Science and Technology, Mirpur, Pakistan; 5 Department of Computer System Engineering, Mirpur University of Science and Technology, Mirpur, Pakistan; 6 School of Computer Science, Faculty of Science and Engineering, University of Hull, Hull, United Kingdom; Khon Kaen University, THAILAND

## Abstract

This paper considers the generic problem of a central authority selecting an appropriate subset of operators in order to perform a process (i.e. mission or task) in an optimized manner. The subset is selected from a given and usually large set of ‘*n*’ candidate operators, with each operator having a certain resource availability and capability. This general mission performance optimization problem is considered in terms of Unmanned Aerial Vehicles (UAVs) acting as firefighting operators in a fire extinguishing mission and from a deterministic and a stochastic algorithmic point of view. Thus the applicability and performance of certain computationally efficient stochastic multistage optimization schemes is examined and compared to that produced by corresponding deterministic schemes. The simulation results show acceptable accuracy as well as useful computational efficiency of the proposed schemes when applied to the time critical resource allocation optimization problem. Distinguishing features of this work include development of a comprehensive UAV firefighting mission framework, development of deterministic as well as stochastic resource allocation optimization techniques for the mission and development of time-efficient search schemes. The work presented here is also useful for other UAV applications such as health care, surveillance and security operations as well as for other areas involving resource allocation such as wireless communications and smart grid.

## 1. Introduction

This paper considers the generic problem of selecting an appropriate subset of **operators** in order to perform a process (**mission/task**). The subset is taken from a given and usually large ‘*n*’ elements set, with each operator having a certain resource availability/capability. The aim, underpinning this selection procedure, is the successful and efficient completion of the mission. This is essentially a discrete optimization problem which requires knowledge of: i) a corresponding **mission/task model** (MM), ii) a mission performance objective measure (MPOM) and iii) the resources available to operators and also associated resource related constraints.

Furthermore in order to study such a generic problem, in conjunction with a real application scenario, a firefighting mission paradigm has been adopted. In this case the above optimization problem is addressed in terms of the deployment of Unmanned Aerial Vehicles (UAVs) acting as firefighting operators in a fire extinguishing mission. UAVs which can be deployed from a single or from multiple geographical positions, are therefore required to deliver fire extinguishing material(s) at the appropriate fire incident location (FL). Thus the selection of a subset of available UAVs in a way that maximizes mission benefit (performance), given certain resource related constraints, is effectively the nonlinear, binary variables optimization problem whose efficient solution is the underlining theme of the paper.

The binary variables resource allocation optimization problem outlined above can be stated as:

$$\begin{array}{c} \begin{array}{c} Minimize\;\;\;\;\;F(X)\\ Subject\;to:\;\;AX\leq Q\\ X\in {\left(0,1\right)}^{n} \end{array} \end{array}$$ (1)

where **F** is a real valued, continuous and non differentiable function, **X** is the vector whose binary valued elements represent the absence (i.e. = 0) or participation (i.e. = 1) in the mission for each of the *n* available operators, **A** is a resource related matrix and **Q** is a vector representing resource constraints.

In general, the problem of Resource Allocation (RA) can be addressed in a number of different ways. These may differ in the presentation of input data, the handling of constraints, the search method used for finding the best solution and the computational complexity and robustness of the optimization approach. There are also hybrid instances available in the literature of scenario-based presentations of input data. Mulvey, Vanderbei and Zenios [1], for example, produced an approach that considers optimization formulations with a scenario-based description of input data. Each scenario is associated with a set of possible instances of uncertain problem data and with the probability of occurrence of that scenario. The authors use this approach to enhance optimization robustness. Furthermore, they devised a model to measure the trade-off between algorithmic optimization quality and robustness. In [2], Kouvelis and Yu present a more detailed account of scenario-based robust optimization techniques.

Deterministic optimization methods consider all the input variables and the resulting mission benefit as deterministic quantities whereas in stochastic optimization techniques, some of the input variables and hence the mission benefit are subject to uncertainty. In the second case, optimization robustness is of fundamental importance and is sought against variable uncertainty and worst case scenarios. A detailed account of deterministic optimization techniques is presented in [3]]. Note that variable and mission benefit uncertainty is a common feature of real applications where the use of resource allocation procedures is required. Various methods of implementing stochastic optimization techniques can be found in [4]. Robust stochastic optimization methods, other than scenario based techniques have been considered by Bertismas and Sim in [5].

A number of approaches have been developed for solving discrete mixed integer programming problems, via their transformation into continuous problems. These include global optimization techniques, semi-definite programming and spectral theory [6–8] and are particularly useful when the discrete to continuous transformations do not affect optimum or near optimum solutions.

Direct discrete optimization can be achieved using: i) Heuristic or ii) Exhaustive search techniques and clear tradeoffs between computational complexity and quality of optimization exist. Exhaustive Optimization (EO) evaluates all possible combinations of decision variables and the best solution that also satisfies resource constraints, is selected. Discrete EO is NP-hard [9, 10] and provides globally optimum solutions, but is computationally expensive and often intractable when the number of decision variables becomes large. Heuristic search algorithms are application specific and they are often employed to quickly produce suboptimum but “good enough” solutions. Using such Heuristic methods, decision variables can be selected on the basis of one or more heuristics and constraints. Examples of Heuristic search techniques can be found in Graph Theoretic [11] methods. Other popular search approaches include Simulated Annealing [12], Genetic Algorithms [13] and Scatter Search techniques. A detailed account of Heuristic type search techniques can be found in [14–17].

In this paper, both Suboptimal Heuristic and Optimal Exhaustive discrete non-linear combinatorial optimization methodologies with linear constraints are considered with respect to deterministic and stochastic input variables. Whereas the deterministic optimization employed in the case of deterministic input variables is conceptually straightforward, the adopted stochastic approach used in the case of stochastic variables is based on the introduction of penalty terms in the calculation of mission performance.

Thus several discrete optimization techniques are developed and their performance characteristics examined within a firefighting mission driven framework. Computer simulation based experimental results allow tradeoffs between computational complexity and quality of optimization to be identified and thus for the performance of these methods to be effectively compared.

The novel scientific contributions of this research work are summarized as follows.

1. The main contribution of the paper is development of novel optimization techniques for resource allocation in UAVs firefighting mission in both deterministic and stochastic scenarios. The optimization techniques include exhaustive search as well as innovative and efficient heuristic search methods.
2. In order to achieve the above-mentioned main contribution, novel UAV firefighting mission framework and Mission Performance Objective Measure (MPOM) have been developed.

Task allocation among UAVs has been considered previously with different objectives such as enhancement in wireless communication networks, see for instance [18–21]. The research work presented here, models a firefighting mission to demonstrate the generic resource allocation problem which is applicable to a broad range of scenarios including the wireless networks resource allocation.

**Fig 1:**
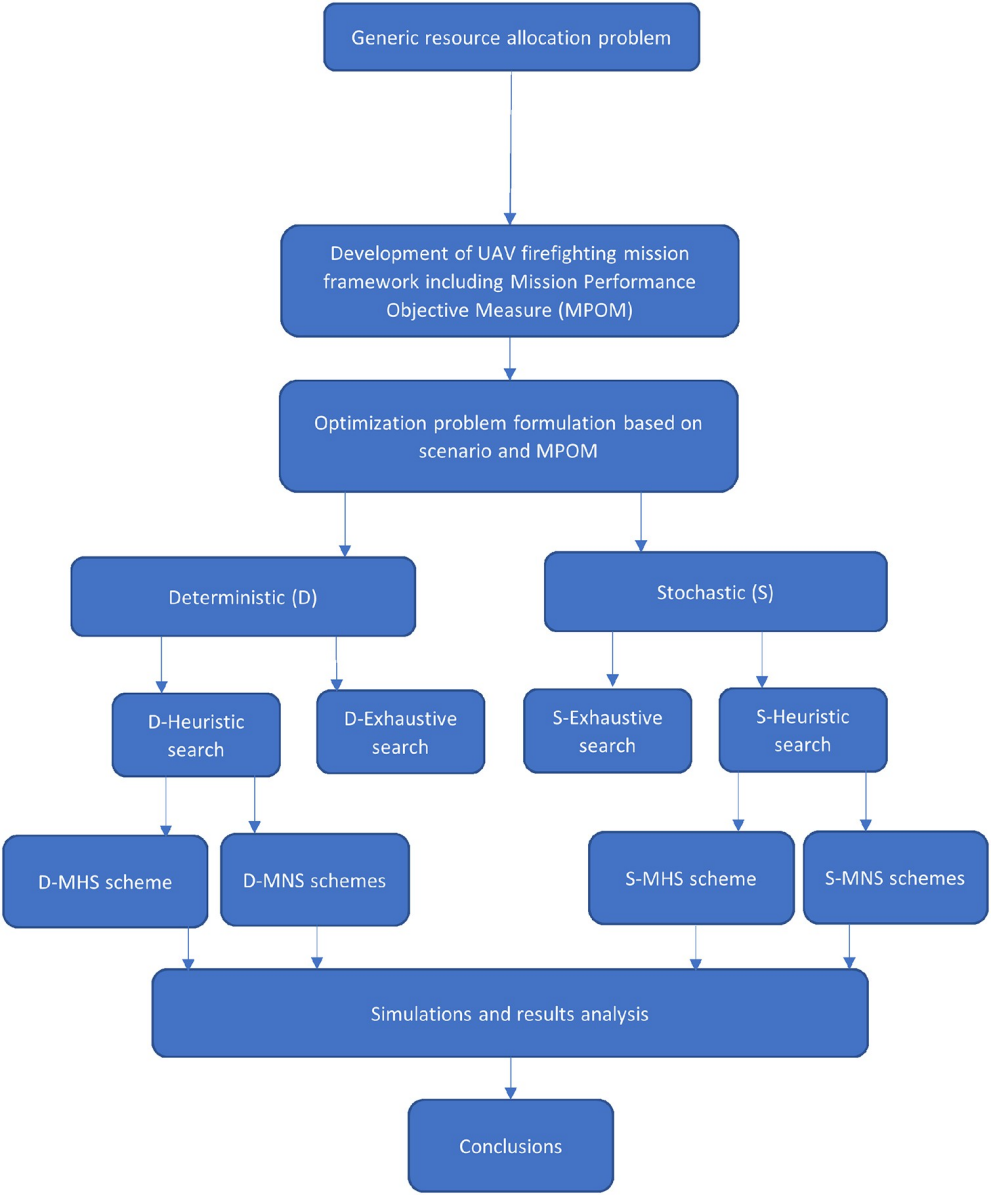
Research flow.

The paper is organized as follows: Section 2 describes the adopted firefighting mission framework that has been employed throughout the work and presents a mission model and corresponding mission performance objective measure to be employed within this framework. It also describes the generic deterministic and stochastic resource allocation optimization methodologies which form the backbone of subsequently proposed efficient optimization schemes. Several sub-optimal optimization techniques are presented in section 3. A simple heuristically driven search optimization scheme is presented in section 3-A, whereas two further multistage optimization schemes are described in section 3-B. The complexity characteristics of the above schemes are considered in Section 3-C. Experimental results are presented in section 4 which allow for a comparative study and appraisal of the proposed methods. The paper concludes with a summary of the proposed schemes as well as a few future work directions. Fig 1 gives overall flow of the presented research work.

## 2. UAV firefighting

Mission (FFM) framework Consider that *n* UAVs are located at *L* different geographical positions (bases) and that they are candidate members of a firefighting mission. Let the amount of the *i^th^* resource (e.g. foam, powder, water etc.) available at base *l* be $Q_{i}^{l}$ and *l* = 1, 2, …, *L* and *i* = 1, 2, …, *m* and the capacity of the *j^th^* UAV located at base *l* to carry resource *i* be $A_{i,j}^{l}$ *j* = 1, 2, …, *n*_*l*_ and that *n*_1_ + *n*_2_ + … + *n*_*L*_ = *n*. The above *m* mission related resources are made of materials which a UAV can carry and deliver for fire suppression e.g. foam, water etc. In addition there are UAV “operational” types of resources that allow (or otherwise) a UAV to reach the Fire Location (FL) e.g. fuel capacity, operational (communication) range etc. Thus UAVs (operators) are of different types and capabilities, as related to their speed, fuel consumption and fire extinguishing resource capacity.

**Fig 2:**
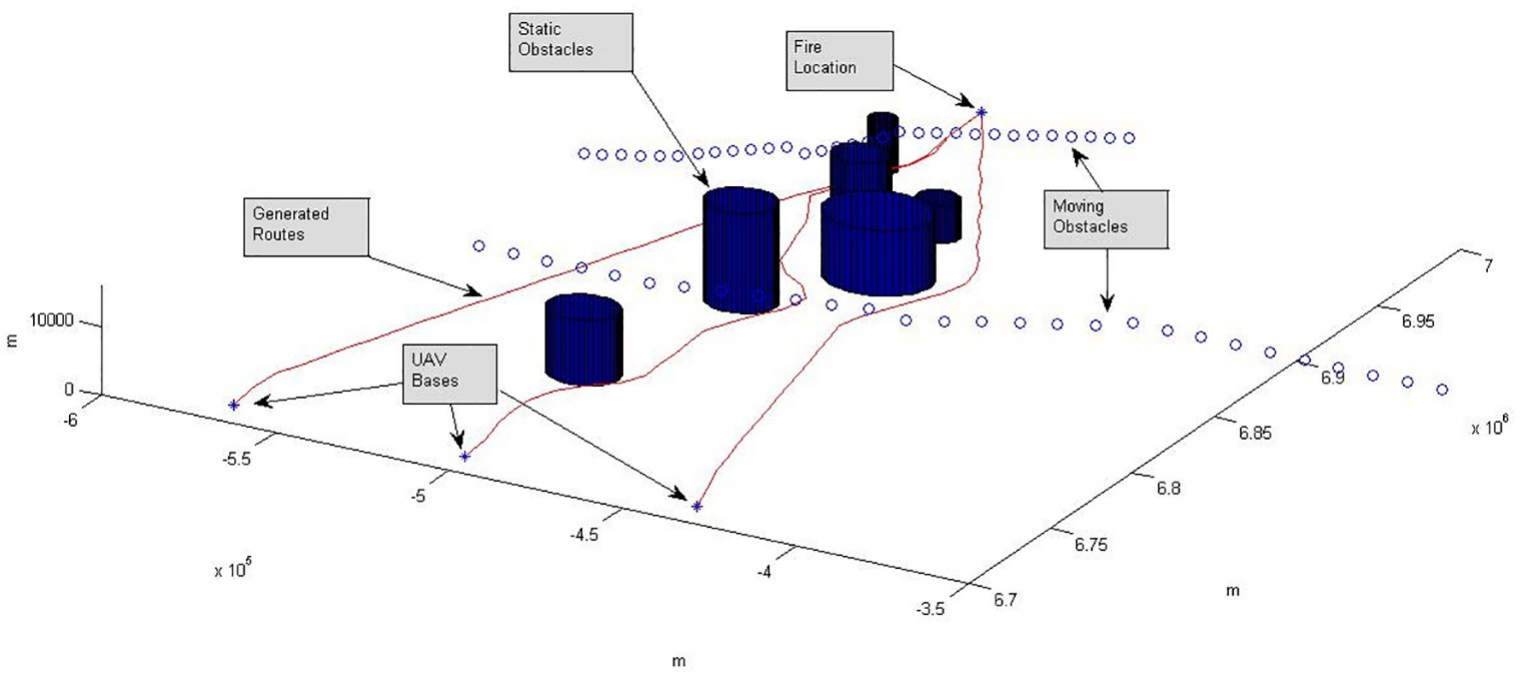
Router calculates three UAV routes commencing from three UAV locations (bases) to a single fire area, while avoiding six static hazards and two moving objects. The work in [23] uses geodetic coordinates for route generation which is converted to Cartesian coordinates for simplicity of calculations and resulting plots. The interested reader may refer to [23] for further details. All axes show length in meters.

Now given the locations of UAV Bases (BL) and FL, an initial per UAV route planning process [22, 23] is performed to obtain *n* base-to-fire (plus fire-to-base) route paths (flight distances *D*_*j*_, *j* = 1, 2, …, *n*) see Fig 2. Thus given route trajectories and based upon specific UAV speed operational profiles, estimates can be obtained for times *t*_*arrival*_ of UAV arrivals to FL, flight time during resource delivery over FL, overall flight times *T*_*M*_ and expected fuel consumptions *F*_*c*_. In practice UAVs tend to fly most of their routes at relatively constant speed and altitude and the models used to calculate fuel consumption and time of arrival can vary significantly in terms of complexity and accuracy, from simple to quite sophisticated and accurate. Since however these models exist and are independent of the optimization techniques examined in this paper, they will not be further pursued. Same applies to the control methodologies used across UAV platforms. The interested reader may refer to [24–28] for an insight into UAV control methodologies.

Note that, in general, operational type of resources act as a “switch” that determines UAV inclusion or exclusion from the optimization process. For example, UAVs whose fuel resource (supply/capacity) is less than *F*_*c*_ giving rise to an overall flight time that is less than the required time *T*_*M*_ cannot safely contribute to the mission and are therefore excluded from the following UAV selection/optimization process.

Now recall that essential to the optimization process is the development and use of a Mission Model (MM) and associated Mission Performance Objective Measure (MPOM). MM and MPOM are application specific and, in practice, key to the success or otherwise of the mission driven resource optimization.

### A. Mission model and mission performance objective measure

In this work MM and MPOM are based on Fire Intensity *FI* that is a function of time. In turn FI(t) can be modelled as a linear or nonlinear function of time *t*, where

$$\begin{array}{c} FI\left(t\right)=\frac{1-b}{{\left[max(t)\right]}^{z}}t^{z}+b \end{array}$$ (2)

Note that z = 1 provides a linear model. b is an initial *FI* value.

The model is supported by a certain fire intensity threshold value *FI*_*th*_. When FI(t) becomes larger than *FI*_*th*_, extinguishing materials will have no effect in the sense that fire damage is so extensive that further fire suppression effort is not recommended.

The application of fire suppression materials at specific times will lead into decreasing fire intensity values. Again this effect can be modelled as a linear or nonlinear decrease of Fire Intensity (*FI*) over time. Here however the linear or nonlinear decrease of *FI* is a function of the amount (in appropriate units) of the specific suppression material applied to the fire. Different material types have different rates of fire suppression over a given period of time. Furthermore the time *t*_*suppress*_ allowed over which *FI* suppression is calculated is considered here to be fixed. Thus different types and amounts of fire suppression resources have different *FI* decrease rate profiles over the *t*_*suppress*_ period. Note that after this time period, *FI* will start increasing again unless the *FI = 0* fire state has been achieved. Finally in this model when multiple fire suppression deliveries occur within a period that is less than *t*_*suppress*_, the overall *FI* suppression effect is calculated over a period of time T commencing from the time of the first delivery *t*_*arrival*_ to the time of the last delivery plus *t*_*suppress*_. Such multiple *FI* suppression effects, due to multiple resource deliveries are therefore accumulated over the T period. A simple way to implement this accumulation effect and thus define the reduction (*FI*_*R*_) in *FI*, per unit of time, as a result of the delivery say of *u* different resources each having *a*_1_, *a*_2_,…., *a*_*u*_ units of material, while assuming that a linear *FI* suppression model is employed, is

$$\begin{array}{c} {FI}_{R}=K_{1}\sum_{i=1}^{u}c_{i}a_{i} \end{array}$$ (3)

**Fig 3:**
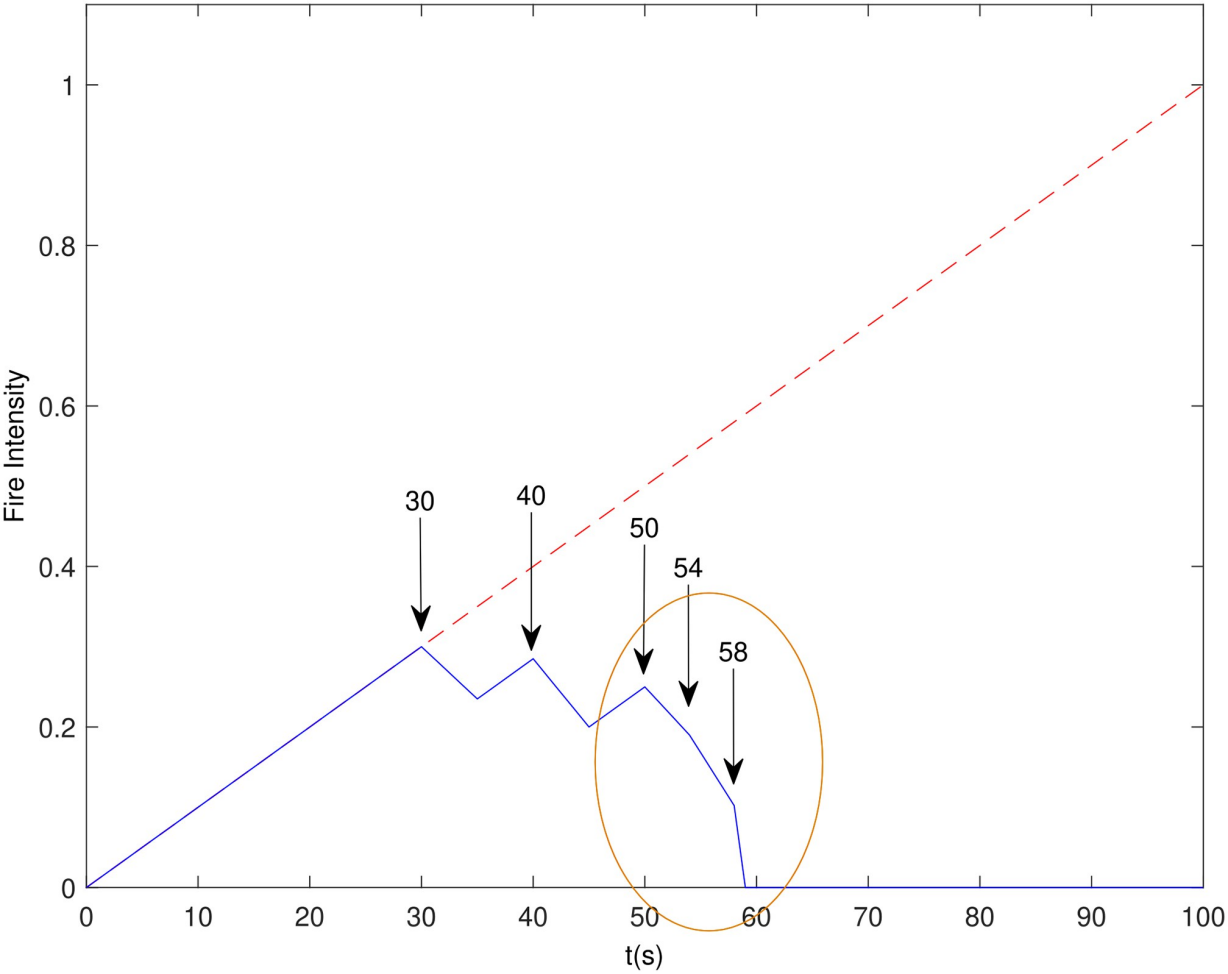
Linear *FI* model used in firefighting mission. Resource delivery times are indicated by arrows. *t*_*suppress*_ = 5 *seconds*.

The *c*_*i*_ coefficients are representative of the effectiveness of different fire suppression materials while *K*_1_ is a normalizing constant. A diagram illustrating FI(t) reduction when multiple fire suppression deliveries occur, is shown in Fig 3. The “broken” red line extension of the blue model line is indicative of the linearly increasing FI(t) without applying fire suppression. The values of related parameters are given in S2 Appendix, section B1.

Note that multiple fire suppression deliveries have been modeled in a generic way. The model uses only two parameters: i) Amount of resources ii) Effectiveness of resources in fire intensity reduction. It means that the model needs to know only amount and effectiveness of a given resource and not its name. For the sake of simplicity, same reduction effect, as given in (3), has been used for all three resources. In Fig 3, an example scenario has been considered, where multiple UAVs arrive for firefighting at times (*t*_*arrival*_) given in the S2 Appendix, section B1 along with matrices A and Q. Each column of matrix A gives amount of three different suppression resources allocated to a UAV, while each row gives allocation of a single type of resource to 10 UAVs. Note that in S2 Appendix, section B1, L = 3 indicates that UAVs are present in three different bases. In each row of matrix A, UAVs 1–4 (*n1*) belong to base 1, UAVs 5–7 (*n2*) belong to base 2 and UAVs 8–10 (*n3*) belong to base 3. The matrix Q gives total amount of three resources available to UAVs at each base, with each row indicating total amount of three resources present at a certain base. Each delivery reduces the fire for 5 *seconds*. When fire suppression due to one delivery is in progress and the next delivery arrives (within 5 *seconds* of the first delivery) then the effect of two deliveries gets combined according to (3). Hence, at times 54 and 58 *seconds*, the rate of fire intensity reduction further increases and the fire state *FI = 0* is achieved.

The MM model described in this firefighting application also provides a means for formulating the required MPOM. Here MPOM has been implemented as

$$\begin{array}{c} MPOM=\int Fl(t)d(t)\; \end{array}$$ (4)

i.e. the area under the FI(t) curve, see Fig 3.

The sooner the fire is extinguished, the smaller is this area and therefore the smaller is the extent of damage caused by the fire. Thus the UAV resource allocation (RA) optimization process can be driven by the minimization of this MPOM objective.

UAV firefighting mission framework includes Mission Performance Objective Measure (MPOM) as given in (4). The MPOM depends on area under the fire intensity curve, see Fig 3 and Eq (2), and is used in both deterministic and stochastic optimization formulations, which are given in the subsequent subsections.

### B. Deterministic optimization

The UAV selection/optimization problem that underpins the previously discussed FFM framework can be stated now as **Mission Objective:**

$$\begin{array}{c} Minimize\;F_{x}=MPOM=\int Fl\left(t\right)d\left(t\right)\; \end{array}$$ (5)

$$\begin{array}{c} subject\;to\sum_{j=1}^{n_{l}}\;A_{i,j}^{l}X_{j}^{l}\leq Q_{j}^{l} \end{array}$$

$$\begin{array}{c} with\;i=1,2,\ldots ,m \end{array}$$

$$\begin{array}{c} and\;n_{1}+n_{2}+\ldots +n_{L}=n \end{array}$$

where *X* ∈ [0, 1]^*n*^ with vector $X=X^{1},X^{2},\ldots X^{L}$ and each sub-vector *X*^p^ $\in {\left[0,1\right]}^{n_{p}}$ having *n*_*p*_ binary elements i.e. $X^{p}=\left\{X_{1}^{p},x_{2}^{p},\ldots ,x_{n_{p}}^{p}\right\}$, with $x_{i}^{p}=1$ or 0.

Two issues should be raised with respect to the above deterministic problem formulation and Exhaustive Search solution approach i) this formulation doesn’t reflect the stochastic nature of parameters often encountered in real applications and ii) search complexity increases exponentially with the number *n* of UAVs (operators) since the search space size is 2^n^.

### C. Stochastic optimization

Consider that $A_{i,j}^{l}$ are random variables. This is justified due to the uncertainty that in practice exists of the effect that the delivery of a given fire suppression resource will have on the mission objective *F*_*x*_. For example, uncertainty here is considered as being generated due to variability in resource delivery accuracy. Let us also assume that the probability density functions (PDFs) of the various $A_{i,j}^{l}$ variables are known. Of course different suppression resources will have different PDFs but we have assumed for simplicity that random variables are Gaussian with specific $A_{i,j}^{l}$[mean] and standard deviation $A_{i,j}^{l}$[SD] values.

In this way, for a given resource *i*, the sum of the random variables $A_{i,j}^{l}$ corresponding to this resource and carried by the UAVs participating in the mission from base *l*, is itself a random variable, i.e.

$$\begin{array}{c} V_{i}^{l}=\sum_{k\epsilon \;supp(x_{j}^{l})}A_{i,k}^{l} \end{array}$$ (6)

where *supp*($x_{i}^{l}$) is the set of UAVs from base *l* participating in the mission.

Now given $V_{i}^{l}$ as in (6), resource constraints can be considered with respect to the following expectation,

$$\begin{array}{c} E[V_{i}^{l}-Q_{i}^{l}] \end{array}$$ (7)

Negative valued expectations imply that $Q_{i}^{l}$ related constraints are not violated. On the other hand, positive valued expectations $E{{[V}_{i}^{l}-Q_{i}^{l}]}^{+}$ imply that corresponding solution violates the $Q_{i}^{l}$ condition. The exact derivation of $E{\left[V_{i}^{l}-Q_{i}^{l}\right]}^{+}$ is usually computationally complex and its inclusion within the optimization process is often computationally impractical.

Instead, a penalty term is introduced and applied in the estimation of the *MPOM = F*_*x*_ measure. This means that non-viable solutions will be penalised in the optimization process by adding values to the objective function, which are proportional to the expected value of the magnitude of the constraint violation.

A scheme is therefore employed which estimates $V_{i}^{l}$ by drawing sample values from random sources representing $A_{i,j}^{l}$ variables, with P successive such sample drawing experiments (trials) per resource *i*.

Denoting $A_{i,j}^{l,p}$ as the value of the $A_{i,j}^{l}$ coefficient in the p^th^ drawing sample experiment, 1≤*p*≤*P*, the resulting estimate of the *i^th^* resource constraint violation, given *l* and *i* is:

$$\begin{array}{c} E{\left[V_{i}^{l}-Q_{i}^{l}\right]}^{+}=\frac{1}{p}\sum_{p=1}^{P}[\sum_{j\epsilon \;supp(X_{j}^{l})}{\left(A_{i,j}^{l,p}-Q_{i}^{l}\right)}^{+}] \end{array}$$ (8)

The *MPOM = F*_*x*_ measure used in the proposed stochastic case takes the form

$$\begin{array}{c} \bar{F_{x}}+W\sum_{l=1}^{L}\sum_{i=1}^{m}E{\left[V_{i}^{l}-Q_{i}^{l}\right]}^{+} \end{array}$$ (9)

${\bar{F}}_{x}$ is the mean of the *F*_*x*_ values produced by running a number (say TN) of trial experiments, which estimate *F*_*x*_ deterministically, given that for each experiment the $A_{i,j}^{l}$ values are drawn from resource related random sources with given probability distributions. W is a weighting constant.

Optimization thus takes the form

$$\begin{array}{c} Minimise\;\;\;\bar{F_{x}}+W\sum_{l=1}^{L}\sum_{i=1}^{m}E{\left[V_{i}^{l}-Q_{i}^{l}\right]}^{+} \end{array}$$ (10)

to obtain an *F*_*min*_ value and corresponding *X*_*opt*_ vector.

Again, as in the deterministic optimization case, an Exhaustive Search can be applied to define *X*_*opt*_. The choice of W depends on the nature of the application domain and level of penalty severity required.

Note that any candidate solution vector X has a non-zero probability to be non-feasible by violating the problem constraints. The weight W, as applied on the penalty terms, controls the importance the optimization attaches to the constraints of the problem. Low W values tend to bias the optimization in favour of solutions which are likely to violate these constraints i.e. the probability of the solution being non-feasible, increases. Large W values have the opposite effect.

**Fig 4:**
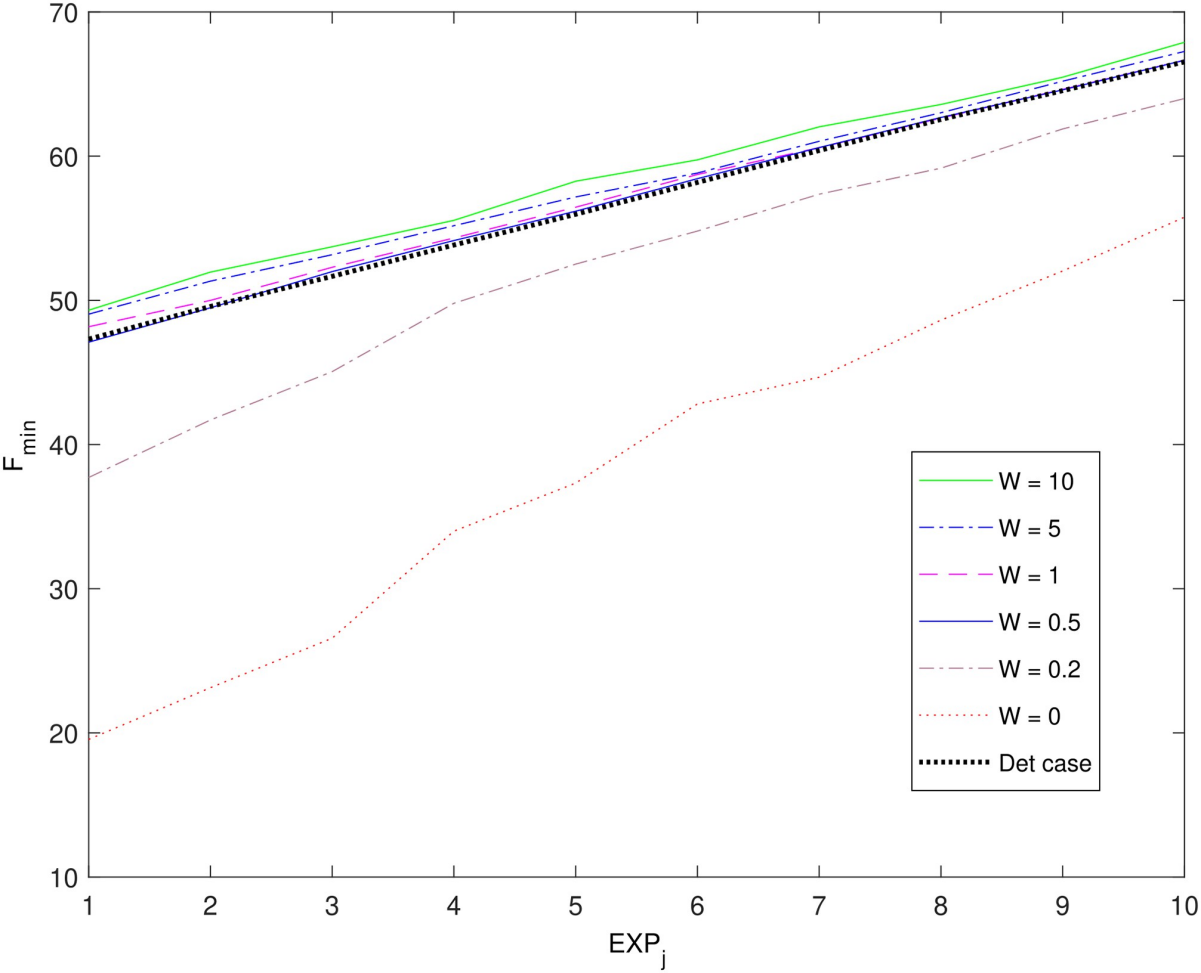
*F*_*min*_ results for 10 different experiments (*EXP*_*j*_), with UAV arrival time values t_*arrival*_ progressively and uniformly increasing for j = 1,2,..,10.

Fig 4 illustrates the effect of W on *F*_*min*_ values obtained by applying the stochastic optimization scheme for 10 different mission scenarios and for different W values. The deterministic optimization case is also included. Mission scenarios have been arranged across the horizontal axis so that UAV arrival time values *t*_*arrival*_ are increasing progressively and uniformly. As W decreases the violation penalty term decreases and in turn the probability of obtaining potentially unsuitable X vectors as solutions with minimum *F*_*x*_ increases. The values of different parameters related to Fig 4 are given in S2 Appendix, section B2.

## 3. Sub-optimum search (SBS) techniques

The Exhaustive Search (ES), which has been mentioned in the deterministic and stochastic optimization methodologies described in previous sections, is optimal and a global minimum solution can always be found. Its complexity however increases exponentially with respect to the size *n* of the vector X and ES becomes impractical for large values of *n*. Thus efficient, in terms of complexity and performance, suboptimal search methods are of considerable interest.

Within the UAV FFM framework the paper considers search efficient techniques, in terms of i) Heuristics, i.e. using application specific information to influence the search and ii) neighbourhood similarity, between vectors X, which can be employed within a Multistage Optimization process to reduce the size of the search area. These suboptimal techniques are indicative of search methodologies which can be formulated and incorporated into the deterministic and stochastic FFM resource optimization approaches. Their development and inclusion here allows a comparative study to be carried out in terms of mission performance versus complexity and possible tradeoffs to be examined regarding different firefighting scenario configurations and strategies.

### A. Multistage heuristic search (MHS)

Heuristic search algorithms have been proposed in different research works for various applications, see for instance [29–31]. The work presented here is generic in nature and may be applied to any similar applications.

In its simplest form, an application specific Heuristic Measure (HM) is calculated, for each operator, that is indicative of the operator’s worthiness to the mission. Consider that a central authority performs the selection across all the UAV bases participating in the mission. UAVs are listed in an ascending (or descending) order from a min (or max) to a max (or min) value of this measure. A multistage UAV selection process then follows whereby in the first stage the UAV at the top of the list is tested for violation of any mission related constraints. A clear i.e. no-violation test result ensures the UAV’s inclusion in the mission and the scheme measures the UAV’s effect on mission performance *F*_*x*_. The second UAV in the list is then considered during the second stage of this process and its inclusion and contribution to *F*_*x*_ is examined, as in the first stage. The process continues until all available for consideration UAVs are examined or a stage is reached where *F*_*x*_ is zero.

A Heuristic measure used in such a simple suboptimal MHS can be based on one or a combination of mission parameters/resources. Thus, for example, HM can be equal to estimated UAV arrival times *t*_*arrival*_ to fire location FL, since an earlier intervention can be thought as beneficial to the minimization of *F*_*x*_. Alternatively HM can be a weighted combination of *t*_*arrival*_ values and $A_{i,j}^{l}$ resource capacity values, for example,

$$\begin{array}{c} {HM}_{j}=w_{1}.t_{arrival}+w_{2}\sum_{i=1}^{M}b_{i}A_{i,j}^{l} \end{array}$$ (11)

where the *w* and *b* weights are constants and

$$\begin{array}{c} w_{1}\;+w_{2}\;=1\;\text{,}\;\sum_{i=1}^{M}b_{i}=1 \end{array}$$

### B. Multistage neighbourhood search (MNS)

The above MHS method examines one operator i.e. UAV, at each stage of the search process and thus formulates sequentially the required mission vector **X**. In contrast, the MNS strategy examines at each stage a relatively small subset of the **X** vectors space. Thus at each stage, say *j*, MNS searches a subset of vectors in order to identify the one vector having minimum *F_*x*_(j)* value. This vector is then used as a “seed” vector to define the next subset to be searched in the following (*j*+1) stage. The process continues for a pre-specified number of stages according to complexity and associated search time considerations. Alternatively, a Measure of Change (MC) of mission performance, that at stage *j* is a function of *k* previous absolute values [*F_*x*_(j) − F_*x*_(j − 1)*] i.e.

$$\begin{array}{c} MC=f(abs(F_{x}(j)-F_{x}(j-i)))\text{,}\;i=0,\;\text{1,}\;\text{2,}\;\text{…,}\;k \end{array}$$

can be defined across successive stages and used as a criterion that allows MNS to terminate automatically when *MC* ≤*ϵ*_*th*_; *ϵ*_*th*_ being a small threshold value. Please note that the indices *i* and *j* used here are specific to this section only and should not be confused with the previously used indices. As the MNS method proceeds across stages, its convergence towards a near optimum solution vector X depends upon i) the initial subset of vectors and ii) the method used to define subsequent subsets. Note that the initial subset of vectors can be selected by sampling the search space i) randomly or ii) by using application specific knowledge. In this work a measure *D(X_*i*_,X_*j*_)* that reflects the similarity between two solutions *X*_*i*_ and *X*_*j*_ has been employed for deriving the next stage subset of possible vectors X. When dealing with binary vectors, a convenient such distance is the Hamming Distance DH. *DH(X_*i*_,X_*j*_)* is equal to the value of the difference between the number of 1s contained in *X*_*i*_ and *X*_*j*_. Thus member vectors of the next, say *j^th^* subset of possible solutions differ from the binary seed vector, which has been identified in the previous i.e. (j-1) stage as having minimum *F*_*x*_, by only a 1 valued element replacing a 0 or conversely a 0 replacing a 1. This MNS scheme, that is effectively based on the similarity condition | *DH(X_*i*_,X_*j*_)* = 1 |, is referred hereafter as MNS-1.

An alternative MNS scheme, namely MNS-2, has also been developed. In this case an initial vector X that contains all 0 elements is used as the initial seed to generate the stage one subset of vectors with | *DH(X_*i*_,X_*j*_)* = 1 |. In all subsequent stages the similarity measure used is | *DH(X_*i*_,X_*j*_)* = 1 | and *DH(X_*i*_,X_*j*_)* < 0. This effectively means that at each stage of the search, say *ST*_*k*_, the subset under examination consists of vectors whose number of vector elements represented by 1, say *STN*_*k*_, is one more than the corresponding number of 1s found in the vectors of the previous stage subset, i.e. *STN*_*k*_ = *STN*_*k*−1_ + 1. MNS-2 can be therefore described as a multistage search where at each stage an iterative search determines the most appropriate (in terms of *F*_*x*_ of the remaining UAVs to be added to the mission. The process terminates when *F*_*x*_ = 0 or when all UAVs have been considered.

### C. SBS complexity characteristics

The main motivation in developing Sub-optimum Search (SBS) techniques is the reduction of search complexity. Recall that the complexity of the optimal Exhaustive Search (ES) grows exponentially with increase in the number of operators *n*. Complexity here is considered in terms of the number of vectors X examined during the search process. Thus with X having size *n*, an Exhaustive Search examines 2^n^ possible solution vectors.

In contrast, the Multistage Heuristic Search (MHS) examines only up to *n* vectors whereas the Multistage Neighbourhood Search -1 (MNS-1) examines up to *Kn* vectors, where *k* is the number of stages adopted by the search. Finally the Multistage Neighbourhood Search-2 (MNS-2) scheme examines up to n(n+1)/2 vectors. Note that all of these SBS schemes have considerably better complexity characteristics (with respect to *n*) than ES.

**Fig 5:**
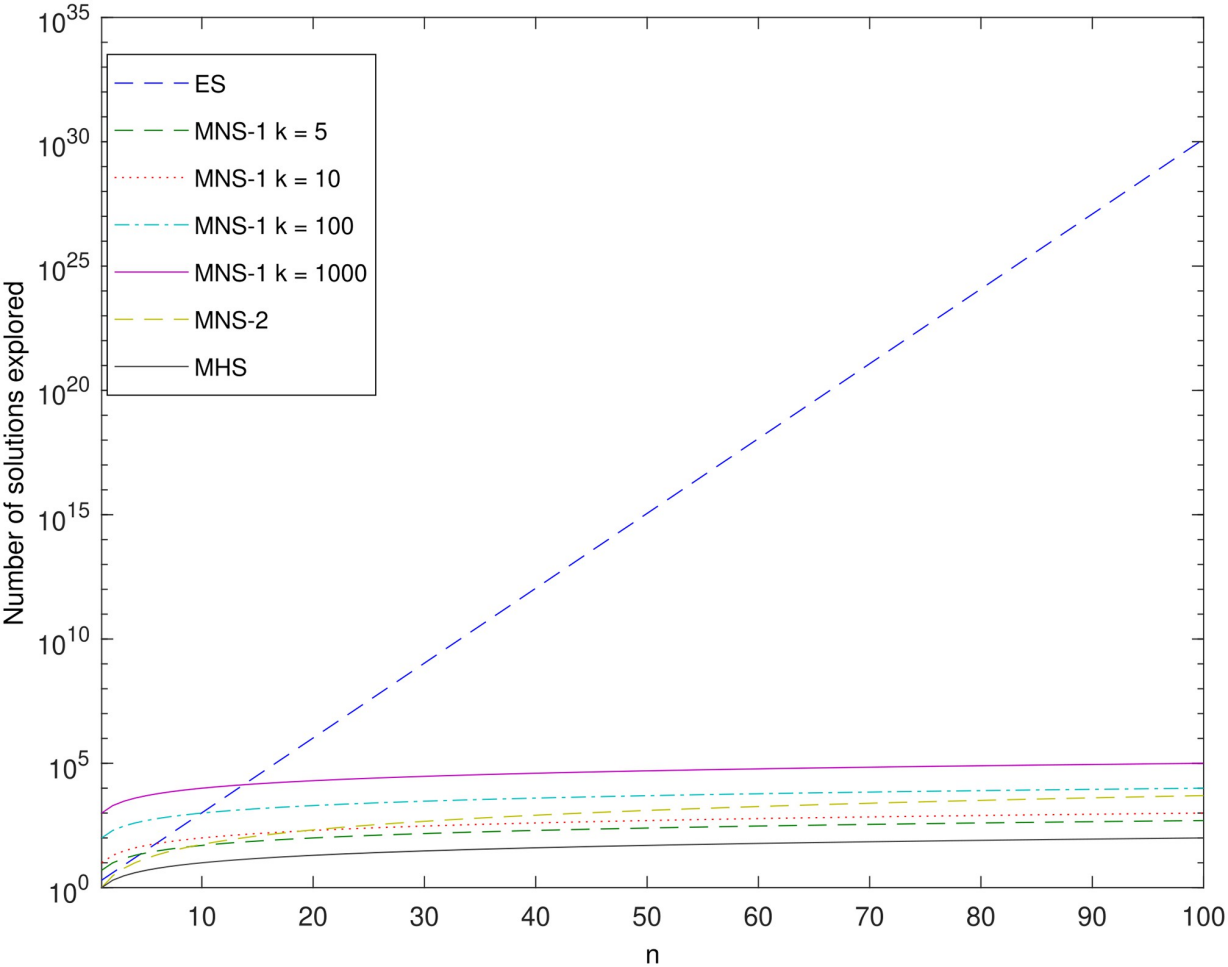
Complexity comparison between ES and SBS schemes.

Fig 5 shows the maximum number of solution vectors X examined by the above schemes, as a function of *n*. i.e. the number of candidate UAVs considered by the search. MHS offers the lowest complexity followed by the MNS schemes. A comparison between MNS-1 and MNS-2 depends on the number of stages K used by MNS-1. Fig 5 seems to roughly indicate that values of K less than 5 allow MNS-1 to be less complex than MNS-2. In fact for a given value of *n* the two schemes have equal complexity when *K* = *K*_*e*_ = [(n+1)/2]. MNS-1 is less complex when *K* < *K*_*e*_.

Note that all search schemes can be applied to both the deterministic and stochastic optimization approaches.

## 4. Experimental results

In order to study and compare the characteristics of the previously described UAV resource allocation methodologies, experimentation has been carried out via computer simulation. The performance of all schemes is considered with respect to achieved *F*_*min*_ values. These are obtained by performing experiments for 10 different firefighting scenarios and by allowing only one resource allocation parameter to vary systematically across scenarios.

**Fig 6:**
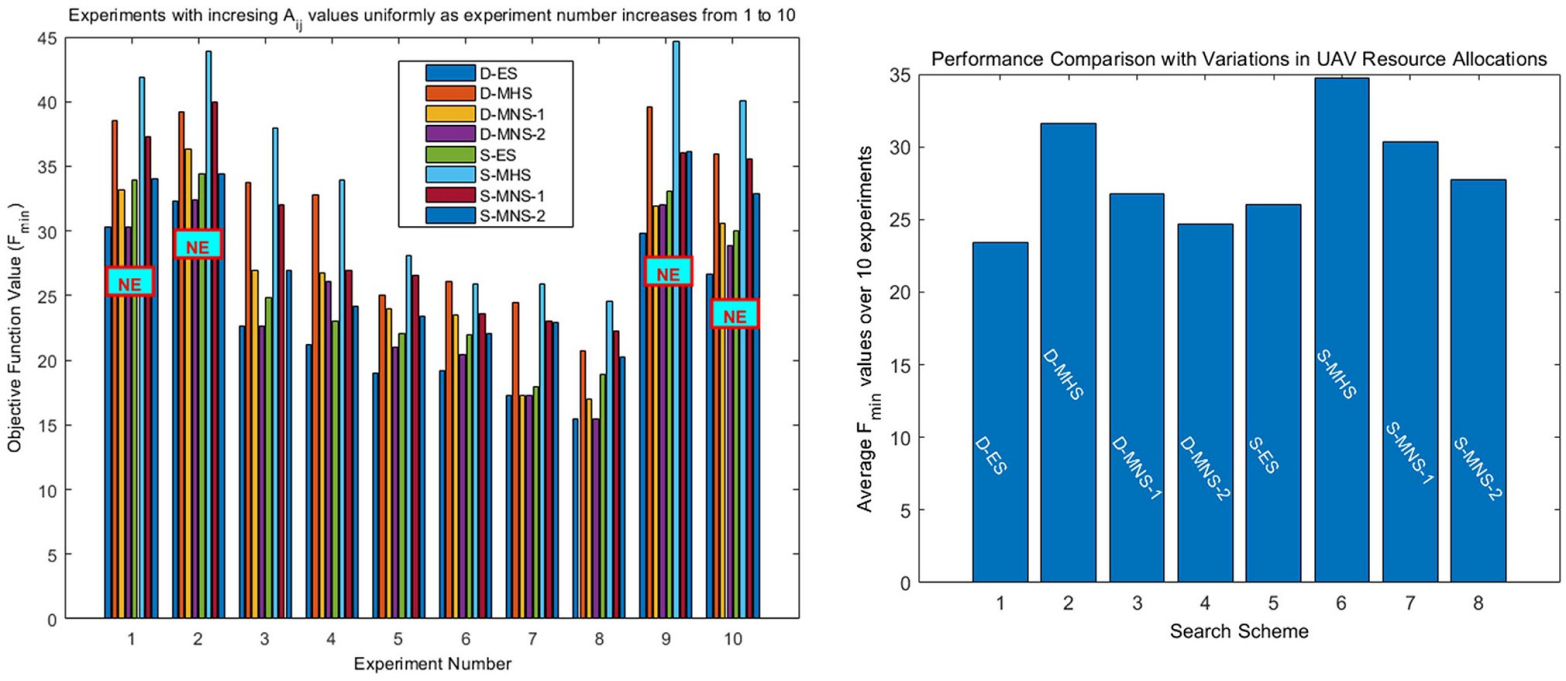
(a) *F*_*min*_ and success/failure results with A_*i*,*j*_ resource values decreasing systematically as experiment number increases from 1 to 10. (b) Performance comparison in terms of average F_*min*_ values for experiments involving variations in allocated resource values. $A_{i,j}^{l}[\text{SD}]=0.1\times A_{i,j}^{l}$ [mean].

Thus Fig 6(a) provides *F*_*min*_ obtained from the D = ES, D-MHS, D-MNS-1, D-MNS-2, S-ES, S-MHS, S-MNS-1, S-MNS-2 methods with scenario parameters defined in S2 Appendix, section B3. On top of *F*_*min*_ values, text box (NE) indicates mission failure (Not Extinguished) while absence of this box indicates mission success (Fire Extinguished). Success implies that the *FI(t)* = 0 state has been achieved. Failure implies that i) at the end of the mission *FI(t)* is not zero or ii) *FI(t)* ≥ *FI*_*th*_. A value of *FI*_*th*_ = 0.9 has been used. Variation between experiments is obtained here by increasing the amount of available resources for individual UAVs i.e. by increasing uniformly the values of all elements in resource matrix A. Note that as fire suppression resource values *A*_*i*,*j*_ increase, *F*_*min*_ values obtained from all methods are not always decreasing. This is due to the fact that increasing resources can violate the resource constraints. In four out of the ten mission scenarios the optimal D-ES scheme fails which implies that all remaining sub-optimum schemes will also fail. In the remaining six missions, all schemes succeed in extinguishing the fire but with a varying degree of success in minimizing fire damage i.e. *F*_*min*_ values. The largest deviation from D-ES *F*_*min*_ values is produced by S-MHS, see experiment 3 (i.e. 15.3055). Also note that in several experiments D-MNS-2 delivers the same *F*_*min*_ values with the optimal D-ES.

It is clear from Fig 6, that the MHS method, although very efficient in terms of complexity, yields *F*_*min*_ which are considerably larger than those obtained from the MNS based methods. This performance behaviour is applicable to both deterministic and stochastic optimization scenarios.

The average *F*_*min*_ values related to Fig 6(a) have been plotted in Fig 6(b), showing average based performance comparison of all deterministic and stochastic schemes, and highlighting again the effectiveness of MNS-2 schemes. The schemes illustrated in Fig 6(a) select a subset of operators (UAVs) for each experiment. In order to elaborate UAV selection in the experiments, Deterministic Exhaustive Search (D-ES) results are opted as specimen. Table 1 shows UAV selection results for ten different experiments with variations in *A*_*i*,*j*_ values. In the first two experiments, only one UAV is excluded by the optimization process governed by (5). Increasing resource quantities *A*_*i*,*j*_ causes two UAVs to be excluded in the third experiment, however remaining UAVs get enough quantities of resources to achieve FI(t) = 0 state. Experiments 3–8 achieve success in the mission, while experiments 9 and 10 fail since a greater number of UAVs are excluded due to constraint violation in (5). Note that a similar mechanism of UAV selection is involved in all the other experiments, although only *F*_*min*_ values and fire suppression status are used to indicate the bahaviour and outcome of the optimization processes. The effect that the standard deviation value, used in the Gaussian PDF of each type of resource, has on the performance of the stochastic search schemes is shown in Table 2. In the first four columns $A_{i,j}^{l}$ [SD] = 0.05 x $A_{i,j}^{l}$ [mean] whereas in the following four columns $A_{i,j}^{l}$ [SD] is set to 2 x $A_{i,j}^{l}$ [mean]. All other system parameters used are the same with those used in Fig 6, see S2 Appendix, section B3. As expected, *F*_*min*_ vary accordingly across experiments and systems as compared to corresponding values in Fig 6. The average objective function values are also given in the last row giving average based performance comparison among all schemes for two different standard deviations.

**Table 1:** UAV Selection obtained from 10 deterministic exhaustive search experiments of Fig 6(a).

Exp. No.	UAV1	UAV2	UAV3	UAV4	UAV5	UAV6	UAV7	UAV8	UAV9	UAV10
1	✔	✘	✔	✔	✔	✔	✔	✔	✔	✔
2	✘	✔	✔	✔	✔	✔	✔	✔	✔	✔
3	✔	✘	✘	✔	✔	✔	✔	✔	✔	✔
4	✔	✔	✔	✘	✔	✔	✔	✔	✔	✔
5	✔	✔	✔	✘	✔	✔	✔	✔	✔	✔
6	✔	✔	✔	✘	✘	✔	✔	✔	✔	✔
7	✔	✔	✔	✔	✘	✘	✔	✔	✔	✔
8	✔	✔	✔	✔	✘	✘	✔	✔	✔	✘
9	✔	✔	✘	✘	✘	✔	✔	✔	✔	✘
10	✔	✔	✘	✘	✘	✔	✔	✔	✔	✘

**Table 2:** F_*min*_ performance results of stochastic schemes. A_*i*,*j*_ resource values increase uniformly across the 10 experiments and results are given for two different $A_{i,j}^{l}$ [SD] values.

	$A_{i,j}^{l}[\text{SD}]=0.05\times A_{i,j}^{l}$ [mean]				$A_{i,j}^{l}[\text{SD}]=2\times A_{i,j}^{l}$ [mean]			
Exp. No.	S-ES	S-MHS	S-MNS-1	S-MNS-2	S-ES	S-MHS	S-MNS-1	S-MNS-2
1	32.7489 (NE)	41.0255 (NE)	37.1054 (NE)	33.9125 (NE)	34.9217 (NE)	44.0147 (NE)	39.1013 (NE)	36.6873 (NE)
2	33.0231 (NE))	42.5937 (NE)	37.9527 (NE)	34.3619 (NE)	36.2839 (NE)	45.1552 (NE)	40.9016 (NE)	36.3902 (NE)
3	23.0528 (Ext)	37.1037 (Ext)	31.9527 (Ext)	25.8261 (Ext)	26.1054 (Ext)	39.9216 (Ext)	34.4288 (Ext)	28.7377 (Ext)
4	22.0162 (Ext)	32.1558 (Ext)	26.2463 (Ext)	23.5515 (Ext)	25.6015 (Ext)	35.5637 (Ext)	28.1452 (Ext)	26.9257 (Ext)
5	21.2996 (Ext)	26.6233 (Ext)	25.8239 (Ext)	22.1602 (Ext)	26.1052 (Ext)	29.7261 (Ext)	27.9557 (Ext)	27.7618 (Ext)
6	21.2306 (Ext)	24.9928 (Ext)	22.9015 (Ext)	22.0516 (Ext)	22.5637 (Ext)	27.4917 (Ext)	24.9144 (Ext)	23.0133 (Ext)
7	17.1545 (Ext)	25.1028 (Ext)	23.0104 (Ext)	21.5104 (Ext)	18.5068 (Ext)	27.0129 (Ext)	25.4872 (Ext)	20.2612 (Ext)
8	18.0129 (Ext)	23.0129 (Ext)	21.7210 (Ext)	19.6474 (Ext)	20.0149 (Ext)	25.6210 (Ext)	23.8198 (Ext)	21.6635 (Ext)
9	32.5283 (NE)	44.6105 (NE)	34.5244 (NE)	34.1745 (NE)	37.0682 (NE)	48.8129 (NE)	39.5379 (NE)	37.8279 (NE)
10	28.5106 (NE)	39.5735 (NE)	34.1935 (NE)	30.4842 (NE)	33.1537 (NE)	43.9120 (NE)	38.1822 (NE)	35.5941 (NE)
Av.	24.9578	33.6795	29.5432	26.768	28.0325	36.7232	32.2474	29.4862

**Fig 7:**
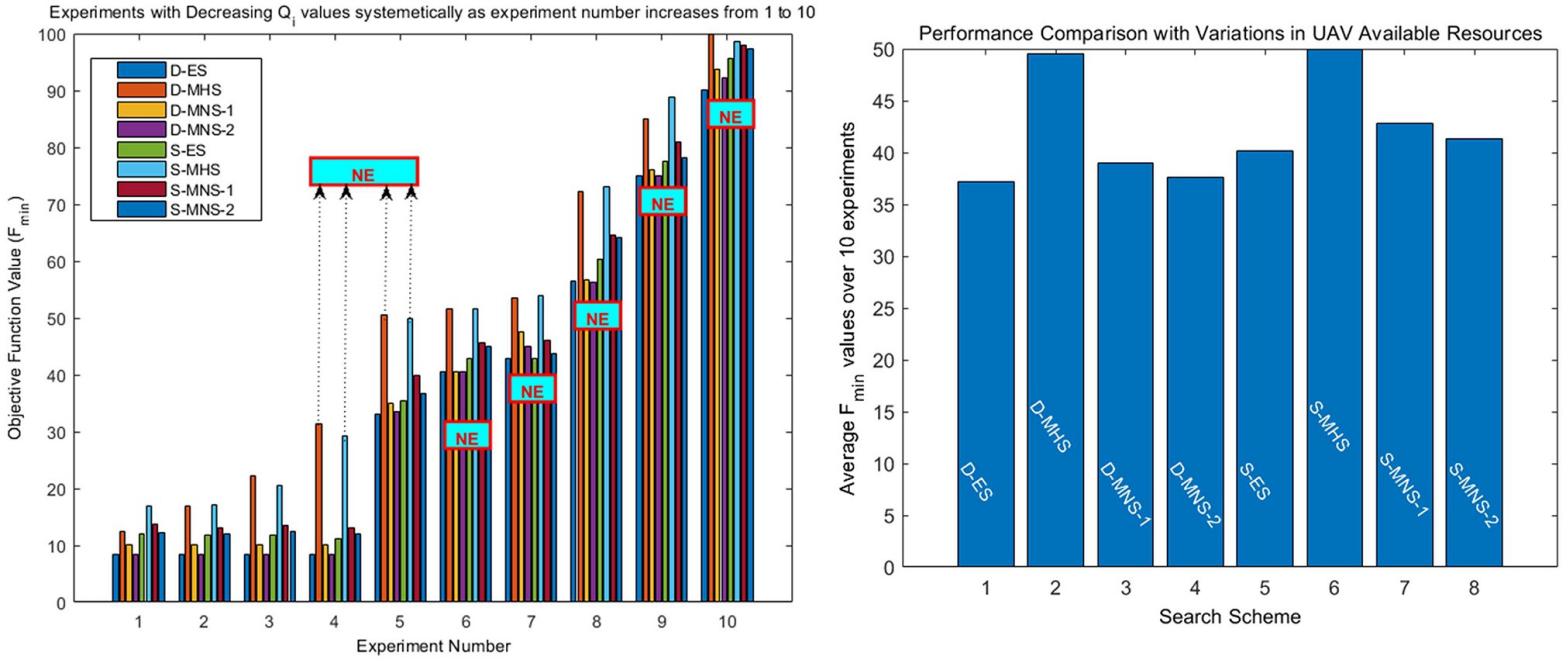
(a) *F*_*min*_ and success/failure results with total resource values decreasing systematically as experiment number increases from 1 to 10. (b) Performance comparison in terms of average values for experiments involving variations in resource values.

Fig 7(a) also presents *F*_*min*_ values and success / failure results for all scenarios and combinations of deterministic/stochastic optimization search methods. System parameters are given in S2 Appendix, section B4. Here however the total value of each resource *Q*_*i*_, that is available in all UAV bases, is decreasing across experiments whereas again all other scenario related parameters remain constant. This type of resource related action amplifies the effect of optimization constraints and as a result *F*_*min*_ tend to increase across optimization scenarios and for all search methods.

The superiority of MNS-2 over MNS-1 is also highlighted here and MHS remains the least effective method. In fact MHS provides a failure result in experiments 4 and 5 (for both D-MHS and S-MHS) whereas all other methods succeed in extinguishing the fire. This has been indicated by a separate text box (NE) along with arrows in Fig 7(a). The accompanying average based performance comparison is given in Fig 7(b).

**Fig 8:**
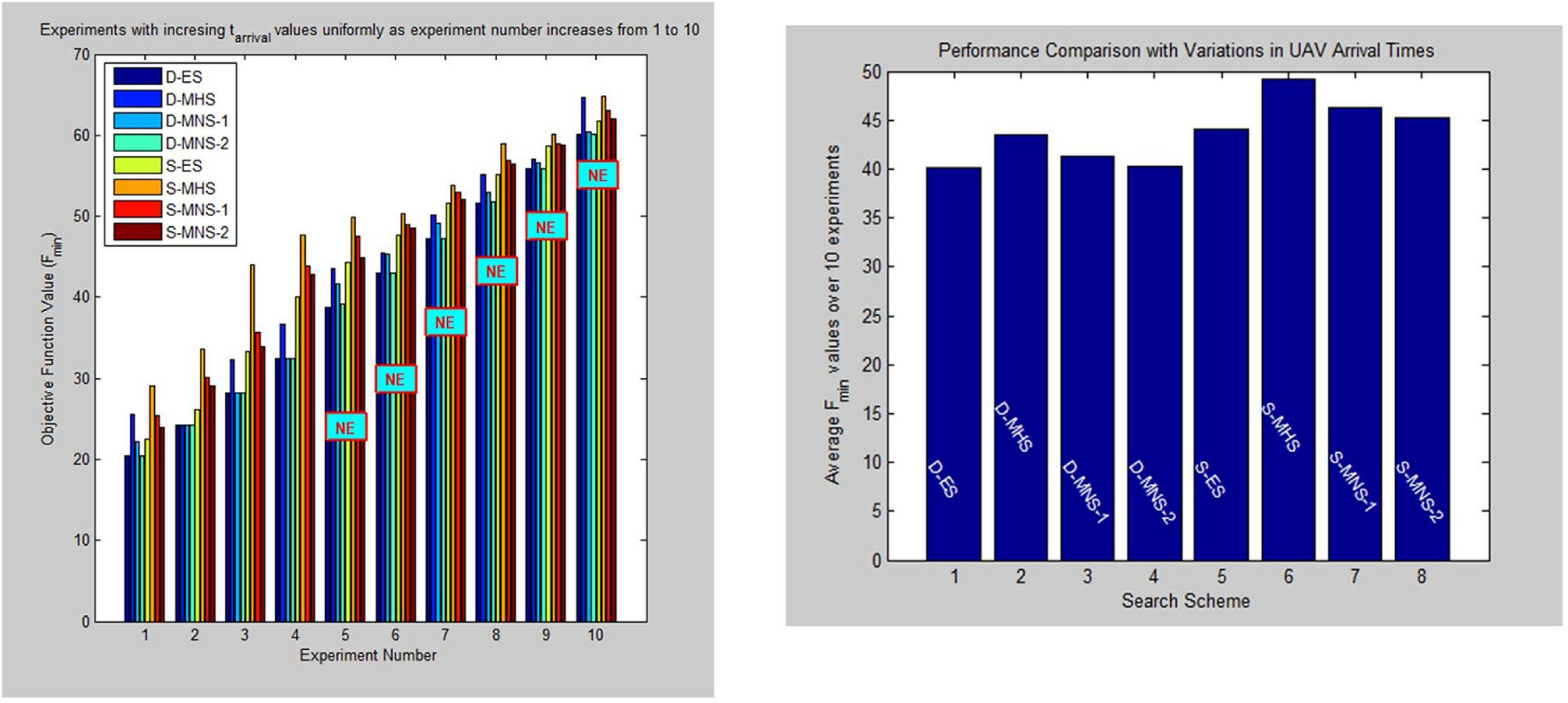
(a) *F*_*min*_ and success/failure results. UAV time of arrival t_*arrival*_ values are increasing uniformly as experiment number changes from 1 to 10 (b) Performance comparison in terms of average F_*min*_ values for experiments involving time of arrival variations.

In a similar way, Fig 8(a) shows the effect that UAV time of arrival *t*_*arrival*_ has on all the above resource allocation schemes. Here UAV *t*_*arrival*_ values are allowed to increase uniformly across 10 experiments. System parameters are given in S2 Appendix, section B5. Note that *F*_*min*_ are increasing as UAV arrival times are progressively delayed and only scenarios 1 to 4 manage successfully to extinguish the fire (indicated by Ext in the Table 2). In all other scenarios the arrival of a number of UAVs is so late that the *FI(t)* > *FI*_*th*_ condition is valid and the mission is declared as failure (indicated by NE in the Table 2). Again in Table 2, *F*_*min*_ values produced by MNS-2 are relatively close to those obtained from the optimal D-ES scheme and offer mission performance advantages over all the other sub-optimal schemes. The same conclusions are drawn by inspecting the accompanying average based performance comparison provided in Fig 8(b).

Finally note that in all of Figs 6–8 experimental results, *F*_*min*_ obtained from schemes involving stochastic optimization are higher than the corresponding values produced by deterministic optimization schemes. This reflects the uncertainty associated with stochastic optimization and the fact that a number of valid and relatively low *F*_*min*_ vectors X can be declared as invalid by the stochastic optimization process.

## 5. Conclusions

The generic resource allocation optimization problem of selecting an appropriate subset of operators in order to perform a given mission or task, has been addressed in this paper in an innovative manner and with algorithmic efficiency and maximization of mission success being the main drivers of this work. Furthermore, and in order to formulate this problem in a realistic and application specific way, the deployment of Unmanned Aerial Vehicles (UAVs) acting as firefighting operators in a fire extinguishing mission framework has been considered and a mission model and associated mission performance measure have been defined. The resulting binary variables resource allocation optimization has been formulated with respect to both deterministic and stochastic input variables. Whereas optimization based on deterministic input variables is conceptually straightforward, the adopted stochastic optimization approach used in the case of stochastic variables is based on the introduction of penalty terms in the calculation of mission performance.

Thus both optimal Exhaustive Search (i.e. D-ES, S-ES) and Suboptimal, Multistage discrete non-linear combinatorial optimization (i.e. D-MHS, S-MHS, D-MNS-1, D-MNS-2, S-MNS-1, S-MNS-1) methodologies with linear constraints, have been developed with respect to deterministic and stochastic input variables.

Exhaustive Search based schemes are particularly complex and impractical when a large value *n* of operators is employed. Suboptimal, Multistage search schemes attempt to provide near optimal performance at a considerably reduced algorithmic complexity. The very simple MHS multistage scheme has shown relatively bad performance as compared to all other schemes (see Figs 6–8) and has been included as an extreme example taken from a possible spectrum of methods with widely varying complexity characteristics. For instance, see Fig 6, where the D-MHS Average *F*_*min*_ is 35.17% higher than that of D-ES. Similarly S-MHS Average *F*_*min*_ is 33.38% higher than that achieved by S-ES. On the other hand the proposed suboptimal Schemes MNS-1 and MNS-2 exhibit both relatively low complexity and a mission performance characteristic that is often slightly worse than or equal (particularly for MNS-2) to that provided by the complex optimal ES schemes.

Note that in all experiments and according to *F*_*min*_ results, the proposed MNS-2 scheme outperforms MNS-1. Average *F*_*min*_ taken from Fig 6 indicate 8.46% and 9.38% inferior performance for D-MNS-1 and S-MNS1 as compared to D-MNS-2 and S-MNS-2 respectively. The corresponding percentages from Figs 7 and 8 are 3.59%, 3.56% and 2.66%, 2.31% respectively.

As a future direction, this work can be extended to communication based fully coordinated system. Some good research articles have already been surfaced demonstrating benefits of communicating UAVs for different applications, see for instance [32–34]. Furthermore, inclusion of UAV flight dynamics and path planning is also suggested in order to enhance the resource allocation work presented here.

## Supporting information

S1 Appendix Nomenclature. (TEX)

S2 Appendix Parameter values used in experiments. (TEX)
